# Association of CD14^+^ monocyte-derived progenitor cells with cardiac allograft vasculopathy

**DOI:** 10.1016/j.jtcvs.2011.07.032

**Published:** 2011-11

**Authors:** Mohamed Salama, Olena Andrukhova, Susanne Roedler, Andreas Zuckermann, Guenther Laufer, Seyedhossein Aharinejad

**Affiliations:** a Department of Cardiothoracic Surgery, Medical University of Vienna, Vienna, Austria; b Laboratory of Cardiovascular Research, Center for Anatomy and Cell Biology, Medical University of Vienna, Vienna, Austria; c Department of Pathophysiology, University of Veterinary Medicine in Vienna, Vienna, Austria

**Keywords:** α-SMA, α-smooth muscle actin, CAV, cardiac allograft vasculopathy, CFU, colony-forming unit, MPC, monocyte-derived progenitor cell, SMC, smooth muscle cell, VEGF, vascular endothelial growth factor

## Abstract

**Objective:**

The pathogenesis of cardiac allograft vasculopathy after heart transplant remains controversial. Histologically, cardiac allograft vasculopathy is characterized by intimal hyperplasia of the coronary arteries induced by infiltrating cells. The origin of these infiltrating cells in cardiac allograft vasculopathy is unclear. Endothelial progenitor cells are reportedly involved in cardiac allograft vasculopathy; however, the role of CD14^+^ monocyte-derived progenitor cells in cardiac allograft vasculopathy pathogenesis remains unknown.

**Methods:**

Monocyte-derived progenitor cells were isolated from blood mononuclear cell fractions obtained from 25 patients with cardiac allograft vasculopathy and 25 patients without cardiac allograft vasculopathy.

**Results:**

Both patients with cardiac allograft vasculopathy and those without cardiac allograft vasculopathy had CD45^+^, CD34^+^, CD14^+^, CD141^−^, CD31^−^ monocyte-derived progenitor cells that differentiated into mesenchymal lineages. Monocyte-derived progenitor cells formed significantly higher numbers of colonies in patients with cardiac allograft vasculopathy than in those without cardiac allograft vasculopathy; this correlated with posttransplant follow-up time. Importantly, monocyte-derived progenitor cells from patients with cardiac allograft vasculopathy expressed significantly more α smooth muscle actin and proliferated at a higher rate than did monocyte-derived progenitor cells of patients without cardiac allograft vasculopathy. In vitro experiments suggested a paracrine control mechanism in proliferation of monocyte-derived progenitor cells in cardiac allograft vasculopathy.

**Conclusions:**

These results indicate that monocyte-derived progenitor cells are associated with cardiac allograft vasculopathy, have the ability to transdifferentiate into smooth muscle cells, and thus may contribute to intimal hyperplasia of coronary arteries in cardiac allograft vasculopathy. Targeting monocyte-derived progenitor cell recruitment could be beneficial in cardiac allograft vasculopathy treatment.

Cardiac allograft vasculopathy (CAV) is the major long-term complication in heart transplant recipients.^1^ With incidences of 8% within the first year, 32% within the first 5 years, and 43% within 8 years after transplant, CAV remains the leading cause of mortality among cardiac allograft recipients despite overall excellent 5-year survivals after heart transplant.^1^ The pathophysiology of CAV is not clearly understood. Although recent work suggests a role for innate and adaptive immune responses,^2^ nonimmunologic factors such as ischemia–reperfusion injury and cytomegalovirus infection may also contribute to CAV pathogenesis.^2,3^

Histologically, CAV is characterized by initial intimal injury of coronary arteries followed by concentric medial thickening as a result of proliferation of vascular smooth muscle cells (SMCs).^4-6^ Consequently, luminal narrowing of coronary arteries develops and eventually results in graft ischemia. Although the mechanisms of intimal hyperplasia are not well understood, evidence suggests that bone marrow–derived multipotent progenitor cells are involved in this process.^7,8^ In this context, recent studies point to the ability of bone marrow–derived progenitor cells to enter the peripheral circulation in response to signals produced after vascular injury.^9^

Many factors have been shown to be involved in progenitor cell mobilization, such as granulocyte colony-stimulating factor, vascular endothelial growth factor (VEGF), and stromal-derived factor 1.^10^ In addition to progenitor cell mobilization, stromal-derived factor 1/ C-X-C chemokine receptor type 4 axis and VEGF have been shown to play key roles in progenitor cell migration to the site of tissue injury.^11,12^

The infiltrating stem cells are thought to differentiate into SMCs,^13^ and in vitro studies suggest that transforming growth factor β and placental-derived growth factor could initiate the differentiation of progenitor cells into contractile SMCs.^14^ There is also evidence that direct cell-to-cell contact between progenitor cells and SMCs can stimulate further differentiation of progenitor cells.^15^ Endothelial progenitor cells have been also thought to contribute to CAV development; however, their role remains controversial.^16,17^ Recent evidence suggests that a decreased level of circulating endothelial progenitor cells is a risk factor for CAV development.^18^ CD14^+^ monocyte-derived progenitor cells (MPCs) are multipotent cells that can be isolated from peripheral blood mononuclear cells.^19,20^ It has been shown that MPCs are characterized by slow proliferation in the absence of growth factor support; expression of the surface markers CD14, CD34, and CD45; and the ability to differentiate into different cell lineages, including osteocytes, chondrocytes, myocytes, adipocytes, and endothelial cells.^19-21^

The role of MPCs in CAV development, however, remains unclear. To address this issue, we initially examined the presence of MPCs in peripheral blood from patients both with and without CAV. Further, the multipotency potential of MPCs was assessed, and their ability to form colony-forming units (CFUs) was compared between the study groups. We have suggested that MPCs might contribute to intimal thickening of coronary arteries of CAV allografts; the expression of α smooth muscle actin (α-SMA) and the proliferation capacity of MPCs were therefore studied. In line with this theory, the presence of MPCs in the media of coronary arteries was studied histologically. Finally, we tried to explore the mechanism by which MPCs are governed.

## Materials and Methods

### Patients and Blood Sampling

This study was approved by the ethics committee of the Medical University of Vienna, and a total of 50 heart transplant recipients who either had CAV (n = 25) or showed no evidence of CAV (n = 25) and gave informed consent to be enrolled were included. Patients were matched for age, sex, cardiac risk factors, and therapy. Table 1 summarizes the demographic data, the most important clinical and hemodynamic characteristics, and the immunosuppressive treatments of study patients. Twenty healthy volunteers who were comparable with study patients with respect to age and sex served as control subjects. In each individual, 20 mL peripheral blood was obtained. One half of the blood was immediately centrifuged to obtain serum, then coded and snap frozen; the other half was processed for mononuclear cell isolation.

### Diagnosis of CAV

CAV was diagnosed with coronary angiography and intravascular ultrasonography. On coronary angiography, CAV was defined as any evidence of luminal irregularities. Meanwhile, reduction of the luminal diameter more than 50% was considered to represent significant stenosis.^22^ Further, the maximal intimal thickening was measured by intravascular ultrasonography, and CAV was defined as an intimal thickness greater than 0.5 mm, as described previously elsewhere.^23^

### Immunosuppression

Immunosuppression of the study patients included methylprednisolone and induction therapy with polyclonal antithymocyte globulin (Table 1). Maintenance immunosuppression was with either cyclosporine (INN ciclosporin; Sandimmun Neoral; Novartis, Basel, Switzerland) or tacrolimus (Prograf; Astellas Pharma, Deerfield, Ill) in combination with mycophenolate mofetil (Cell Cept; Hofmann-La Roche, Grenzach-Wyhlen, Germany) and steroids or cyclosporine in combination with everolimus (Certican; Novartis) and steroids.^24^

### Cell Isolation and Culture

The peripheral blood mononuclear cells were isolated by density gradient centrifugation with Histopaque-1077 (Sigma-Aldrich Corp, St Louis, Mo) according to the manufacturer’s protocol. Peripheral blood mononuclear cells were suspended in low-glucose Dulbecco modified Eagle medium containing 10% fetal calf serum (Gibco; Life Technologies Corporation, Carlsbad, Calif), 50-U/mL penicillin, and 250-μg/mL streptomycin; seeded on 10-μg/mL fibronectin-coated 6-well plates (10^7^ cells/well); and incubated. Under daily observation, first culture medium change was performed after 4 to 7 days; thereafter, medium was changed every 3 days. Adherent fibroblastlike cells were collected at 7 to 10 days as described elsewhere.^20^ The number of CFUs was documented for each patient on day 12 and then repeated daily.^16,25^ Adherent cells staining positively for CD14, CD45, and CD34 were considered to be CD14^+^ MPCs. On reaching 70% to 90% confluence, the cells were washed with phosphate-buffered saline solution, removed from the culture dish by 0.02% trypsin and 200-nmol/L ethylenediaminetetraacetic acid (Invitrogen; Life Technologies) for 5 minutes, and expanded through successive passages.

### Lineage Induction and Analysis

Multipotency of the MPCs isolated from patients with CAV (n = 10) and without CAV (n = 10) was tested with stem cell differentiation kits for adipocyte, chondrocyte, and osteocyte lineages according to manufacturer’s protocol (Invitrogen; Life Technologies). Briefly, MPCs were incubated with the differentiation medium at 37°C for 3 weeks, and culture medium was changed every 3 to 4 days. Foci of mineralization in osteocytes were visualized in cells fixed with 4% formaldehyde by staining with 2% alizarin red dye (Sigma-Aldrich). Proteoglycan synthesis was visualized in fixed chondrocytes with alcian blue in 0.1-N hydrochloric acid (Sigma-Aldrich). Lipid droplets were visualized in adipocytes fixed with 60% isopropanol with oil red O staining (Sigma-Aldrich).

### Proliferation Assay

MPCs (10^4^ cells/well) were seeded to a 96-well plate in 100-μL/well Dulbecco modified Eagle medium supplemented with 10% fetal calf serum. MPCs from patients with or without CAV were starved in serum-free Dulbecco modified Eagle medium for 2 hours, and the medium was then replaced with conditioned medium obtained from corresponding passage of MPCs from patients with or without CAV. After 24 or 48 hours of incubation, cells were incubated in proliferation reagent WST-1 (10 μL/well; Roche Diagnostics, Indianapolis, Ind) for 2 hours before the absorbance at 450 nm was measured with a microtiter plate reader.

### Flow Cytometry

MPCs were stained with phycoerythrin- and fluorescein isothiocyanate–conjugated monoclonal antibodies against CD14 (ImmunoTools, Friesoythe, Germany), CD31, CD34, CD45, CD141 (BD Biosciences; Becton, Dickinson and Company, Franklin Lakes, NJ), CD133 (Miltenyi Biotec GmbH, Bergisch Gladbach, Germany), CD146 (Millipore Bioscience Research Reagents, Temecula, Calif), and VEGF receptor 2 (Research & Diagnostics Systems, Inc, Minneapolis, Minn).^26^ Before staining with α-SMA antibody (Research & Diagnostics Systems), MPCs were treated with a cell permeabilization kit (AN DER GRUB Bio Research GmbH, Kaumberg, Austria) according to the manufacturer’s protocol. MPCs were then analyzed on a FACscan flow cytometer (BD Biosciences; Becton, Dickinson and Company) with an argon laser tuned to 488 nm. Membrane-compromised cells were excluded with 7AAD (BD Biosciences; Becton, Dickinson and Company). The appropriate isotype identical antibodies served as negative controls.

### Immunofluorescence Staining

Paraffin sections of cardiac biopsy specimens (n = 3) obtained from explanted hearts of patients with CAV undergoing cardiac retransplant were blocked in phosphate-buffered saline solution supplemented with 5% horse serum and stained with monoclonal rabbit anti–human CD34 (Abcam plc, Cambridge, UK) and mouse anti–human CD45 antibody (Abcam). Primary antibodies were detected by incubation with isothiocyanate– and phycoerythrin-conjugated secondary antibodies (Polysciences Inc, Warrington, Pa). Slides were then stained with 4′,6-diamidino-2-phenylindole and analyzed under a fluorescent microscope.

### Statistical Analysis

Parameters were compared between patient groups by χ^2^ test and 1-way analysis of variance (Tukey post hoc test) according to the scale of the variable (categoric or continuous). In the case of skewed data, a nonparametric test (Mann**-**Whitney test) was applied. The associations between number of MPC colonies and time after transplant as well as chemokine serum concentrations were analyzed by the Spearman correlation test. Repeated measurements of MPC number and proliferation with time were examined with the Friedman test and repeated measures analysis of variance (Bonferroni correction post hoc multiple comparison test) according to the distribution of the variables. Statistical analyses were performed with the SAS system for Windows, version 9.1.3, and the Enterprise Guide, version 4.1 (SAS Institute, Inc, Cary, NC). Data are expressed as mean ± SD.

## Results

### CAV in Relation to Procedure and Recipient Characteristics

The patients were matched with respect to age, sex, time to transplant, etiology of cardiac disease, diabetes, hyperlipidemia, and immunosuppression to allow comparison between patients with and without CAV. In this cohort, acute cellular rejection, cytomegalovirus match, graft ischemic time, and panel-reactive antibody levels were not significantly different between the patient groups (Table 1).

### MPC Presence in Peripheral Blood of Patients With and Without CAV

**Figure 1:**
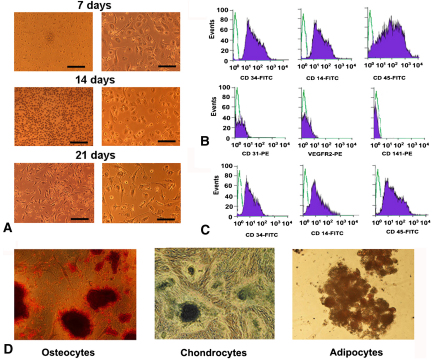
Representative images and characterization of monocyte-derived progenitor cells. A, Light microscopic images of monocyte-derived progenitor cells at 7, 14, and 21 days after initial plating. No cell colony formations were visible at 14 days. Spherical to fibroblastlike phenotype change of monocyte-derived progenitor cells was observed at 21 days. B, Representative flow cytometric staining of low-passage monocyte-derived progenitor cells show stable expressions of CD14, CD45, and CD34 antigens and a lack of CD31 vascular endothelial growth factor receptor 2 *(VEGFR2)* and CD141 antigen expressions. The cells from patients with and without cardiac allograft vasculopathy showed no phenotypic differences in flow cytometric analyses. C, Flow cytometric staining of high-passage monocyte-derived progenitor cells indicate decreased CD34 and CD14 expressions relative to low-passage monocyte-derived progenitor cells. D, Monocyte-derived progenitor cell–derived osteocytes, chondrocytes, and adipocytes stained with alizarin red, alcian blue, and oil red O (*left* to *right*). No differences were observed in differentiation capacity of monocyte-derived progenitor cells when patients with and without cardiac allograft vasculopathy were compared. *FITC,* Fluorescein isothiocyanate.

The cultivated cells isolated from blood of patients with and without CAV formed colonies at 7 days of plating (Figure 1, *A*). Low-passage MPCs of all patients displayed a stable surface antigen expression profile, comprising blood (CD45) and myeloid (CD14, CD34) cell markers but lacking endothelial cell antigens CD141, CD31 and VEGF receptor 2 (Figure 1, *B*). High-passage MPCs in both groups, however, showed lower CD34 and CD14 expressions but unchanged CD45 expression during culture (Figure 1, *C*).

### Multipotency of MPCs Obtained From Patients With and Without CAV

The results of multipotency assays indicated that at both high and low passages MPCs of both patients with CAV and patients without CAV differentiated into osteocytes, chondrocytes, and adipocytes (Figure 1, *D*). These results demonstrate that multipotent CD14^+^ MPCs can be isolated from both patients with CAV and those without CAV.

### Higher Numbers of Colony-Forming MPCs in Patients With CAV

**Figure 2:**
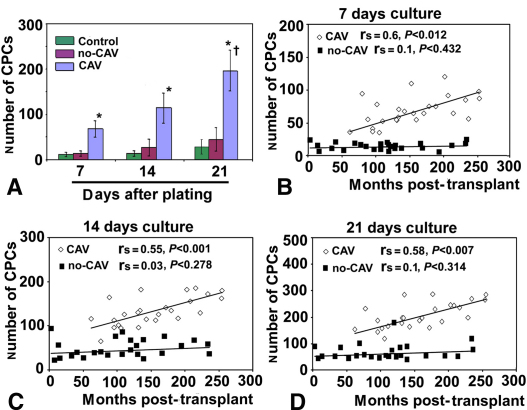
Monocyte-derived progenitor cell *(CPC)* number is associated with cardiac allograft vasculopathy *(CAV)* and correlates with follow-up time since transplant. A, Quantification of monocyte-derived progenitor cell number in patients with cardiac allograft vasculopathy *(CAV),* patients without cardiac allograft vasculopathy *(no-CAV),* and control subjects at 7, 14, and 21 days of plating. B–D, The number of monocyte-derived progenitor cells isolated from patients with cardiac allograft vasculopathy correlates significantly with follow-up time since transplant at 7 days (B; r_s_ = 0.60; *P* < .012). Such a correlation is missing for monocyte-derived progenitor cells isolated from patients without cardiac allograft vasculopathy at 14 days after initial plating (C; r_s_ = 0.55; *P* < .001) and 21 days after initial plating (D; r_s_ = 0.58; *P* < .007). *Asterisk* indicates *P* < .001 versus patients without cardiac allograft vasculopathy and control subjects; *dagger* indicates *P* < .0001 versus 7 and 14 days of plating.

The in vitro assay results indicated significantly higher number of CFUs in patients with CAV than in patients without CAV or control subjects (*P* < .001) at 3 cultivation evaluation time points: 7, 14, and 21 days of plating (Figure 2, *A*). Moreover, repeated measures analysis of variance indicated that the number of CFUs in patients with CAV at 21 days of plating was significantly higher (*P* < .0001) than the numbers of CFUs at 7 and 14 days of cultivation (Figure 2, *A*). Importantly, the number of CFUs in patients with CAV but not patients without CAV correlated with the follow-up time since transplant (Figure 2, *B, C,* and *D*). Specifically, the correlation coefficients for the relationship between the number of MPCs and the follow-up time since transplant in patients with CAV were r_s_ = 0.62 (*P* < .012) at 7 days, r_s_ = 0.55 (*P* < .001) at 14 days, and r_s_ = 0.5 (*P* < .007) at 21 days. In contrast, the correlation coefficients for the relationship between the number of MPCs and the follow-up time since transplant in patients without CAV were r_s_ = 0.1 (*P* < .432) at 7 days, r_s_ = 0.03 (*P* < .278) at 14 days, and r_s_ = 0.1 (*P* < .314) at 21 days. These results indicate that a high number of colony-forming MPCs in vitro is associated with CAV and correlates with the follow-up time since transplant.

### Differential Expression of α-SMA in MPCs of Patients With and Without CAV

**Figure 3:**
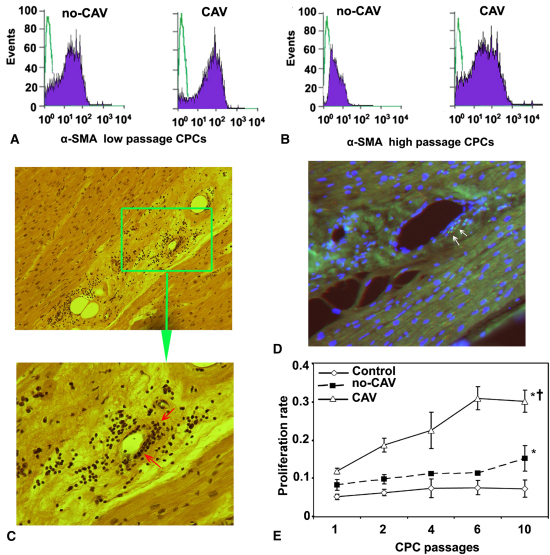
Monocyte-derived progenitor cells *(CPCs)* of patients with cardiac allograft vasculopathy proliferate at a higher rate, express more α smooth muscle actin *(α-SMA),* and are present in cardiac allografts of patients with cardiac allograft vasculopathy. A, Representative flow cytometric analysis indicates that at low (1st) passage, monocyte-derived progenitor cells of both patients with cardiac allograft vasculopathy *(CAV)* and patients without cardiac allograft vasculopathy *(non-CAV)* express α smooth muscle actin independent of cardiac allograft vasculopathy presence or absence. B, At high passage (10th), monocyte-derived progenitor cells from patients with cardiac allograft vasculopathy express α smooth muscle actin significantly more (*P* = .01) than do those isolated from patients without cardiac allograft vasculopathy. C, Hematoxylin–eosin staining shows intimal thickenings and cellular infiltrations *(arrows)* in the media of coronary arteries in patient with cardiac allograft vasculopathy (original magnifications ×16 and ×40 in the *upper* and *lower* panels, respectively). D, Immunocytochemical analysis indicates the presence of monocyte-derived progenitor cells *(arrows)* in the media of coronary arteries in an explanted cardiac allograft of a patient with cardiac allograft vasculopathy (original magnification ×40). E, Monocyte-derived progenitor cells from all study patients, independent of cardiac allograft vasculopathy presence, retain higher proliferative capacity than do those isolated from control subjects. Monocyte-derived progenitor cells isolated from patients with cardiac allograft vasculopathy proliferate at a significantly higher rate at low as well as at high passages than do those isolated from patients without cardiac allograft vasculopathy. *Asterisk* indicates significant difference at *P* < .001 from control for all passages; *dagger* indicates significant difference at *P* < .008 from patients without cardiac allograft vasculopathy for all passages.

Because α-SMA–positive cells are presumed to differentiate into SMCs,^21^ we examined whether MPCs in our patients expressed α-SMA. The analyses in MPCs from patients with and without CAV at low passages showed that MPCs of both patient groups expressed α-SMA, indicating that low-passage MPCs are able to differentiate spontaneously into SMCs (Figure 3, *A*). Only high-passage (8th–10th) MPCs from patients with CAV expressed α-SMA significantly higher (*P* < .01) than did corresponding MPCs isolated from patients without CAV (Figure 3, *B*). These data suggest that MPCs from patients with CAV preserve their ability to differentiate into SMCs even in high cell culture passages.

### Detection of High Proliferation Capacity MPCs in Media of Coronary Vessels of Patients With CAV

Histologic examination clearly indicated concentric intimal thickenings associated with significant cellular infiltration in the media of coronary arteries in explanted cardiac allografts from patients with CAV, and immunocytochemical examination showed MPCs to be a component of these cellular infiltrations (Figure 3, *C* and *D*). The proliferation assay results indicated that MPCs in all patients retained higher proliferative activity independent of CAV presence (*P* < .001) than did those isolated from control subjects, although MPCs isolated from patients with CAV showed significantly higher proliferation rates at both low and high passages (*P* < .008) relative to MPCs obtained from patients without CAV (Figure 3, *E*). Repeated measures analysis revealed that the proliferation rate of MPCs from patients with CAV increased significantly over the culture passages (*P* < .02; Figure 3, *E*); however, Bonferroni post hoc multiple comparison test indicated that only the proliferation rate of MPCs from the 10th passage was significantly different from those of the 1st and 2nd passages (*P* = .033; Figure 3, *E*). There were no significant differences in the proliferation rates of MPCs from patients without CAV (*P* = .068) and control subjects (*P* = .082) when different passages were compared (Figure 3, *E*).

### Paracrine Mechanisms and MPC Proliferation in CAV

**Figure 4:**
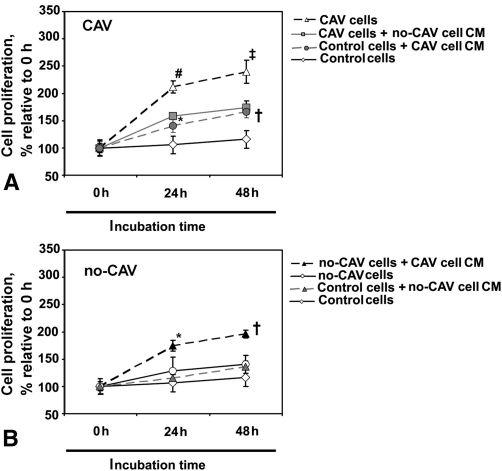
Conditioned medium *(CM)* of monocyte-derived progenitor cells isolated from patients with cardiac allograft vasculopathy *(CAV)* stimulates proliferation of monocyte-derived progenitor cells from patients without cardiac allograft vasculopathy *(no-CAV)*. A, Conditioned medium of monocyte-derived progenitor cells from patients with cardiac allograft vasculopathy significantly enhances the proliferation rate of control monocyte-derived progenitor cells at 24 hours and 48 hours of incubation relative to untreated cells. Additionally, conditioned medium of monocyte-derived progenitor cells from patients without cardiac allograft vasculopathy reduces proliferation rate in monocyte-derived progenitor cells from patients with cardiac allograft vasculopathy relative to untreated cells. B, Conditioned medium of monocyte-derived progenitor cells from patients with cardiac allograft vasculopathy significantly enhances the proliferation rate of monocyte-derived progenitor cells from patients without cardiac allograft vasculopathy at 24 hours and 48 hours of incubation relative to untreated cells. Moreover, stimulation of monocyte-derived progenitor cells from control subjects with conditioned medium of monocyte-derived progenitor cells from patients without cardiac allograft vasculopathy did not change cell proliferation rate relative to untreated cells. *Asterisk* indicates *P* = .02 relative to untreated cells; *dagger* indicates *P* = .04 relative to untreated cells; *hatch mark* indicates *P* = .01 relative to untreated cells; *double dagger* indicates *P* = .03 relative to untreated cells.

To examine the mechanisms by which MPCs gain the ability to proliferate and transdifferentiate into SMCs in patients with CAV, we incubated MPCs from both patients with CAV and patients without CAV in conditioned medium obtained from the counterpart group and subjected the treated cells to subsequent proliferation assays. The results indicate that conditioned medium of MPCs obtained from patients with CAV enhanced the proliferation rate relative to untreated cells of MPCs obtained both from patients without CAV and from control subjects significantly at 24 hours and 48 hours of incubation (*P* = .02 and *P* = .04, respectively; Figure 4). Conversely, treatment of MPCs obtained from patients with CAV with conditioned medium of MPCs isolated from patients without CAV decreased cell proliferation rate relative to untreated cells significantly at 24 hours and 48 hours of incubation (*P* = .01 and *P* = .03, respectively; Figure 4). Stimulation of MPCs obtained from control subjects with conditioned medium of MPCs obtained from patients without CAV did not change the cell proliferation rate relative to untreated cells at both 24 hours and 48 hours of incubation (*P* = .41; Figure 4). These results favor a paracrine control mechanism in proliferation of MPCs in patients with CAV.

## Discussion

Evidence suggests that CAV is the end result of a series of immunologic and nonimmunologic insults to the allograft.^2,3^ Intimal hyperplasia of coronary arteries is the main histologic characteristic of CAV^2^ and develops by accumulation of SMCs and extracellular matrix in a subendothelial location.^27^ This occurs together with infiltration of monocytes, T cells, fibroblasts, and dendritic cells.^27^ The source of the infiltrating SMCs however, remains unclear. In this study, we have provided evidence that MPCs are significantly increased in patients with CAV relative to patients without CAV and migrate into the media of coronary vessels, suggesting an association between MPCs and CAV development. Although the CFU assays used assess adherent cells only, and although immunosuppression can affect the ability of MPCs to adhere, our results allow the comparison of MPCs obtained from the 2 patient groups because they were matched with respect to immunosuppression.

In concert with our results, it has been shown that extracardiac progenitor cells are able to repopulate most cell types in the cardiac allograft.^27^ Moreover, study of SMC chimerism in sex-mismatched heart transplants has revealed that as many as 30% of allograft SMCs in coronary intimas are recipient derived.^28,29^ Further, SMC chimerism in atherosclerotic coronary intimas has been shown to be considerably higher than in nondiseased allografts, suggesting that recipient progenitors might be recruited to the site of allograft injury.^30^ It has been shown previously that immunologic vascular endothelial injury in acute rejection episodes is associated with release of many cytokines and chemokines that stimulate progenitor cell recruitment to the site of injury.^31^ Also, there is evidence that infiltrated T lymphocytes at the site of injury support the migration and differentiation of MPCs.^32^ Accordingly, animal studies indicate that chemokines specific for macrophages and T lymphocytes correlate with mononuclear cell infiltration and precede intimal thickening in CAV.^33^ Our study, in which the treatment of MPCs with conditioned medium of MPCs obtained from patients with CAV enhanced cell proliferation rate, expands our knowledge regarding the role of progenitor cells with respect to CAV pathogenesis and indicates that in addition to the well-known cell-to-cell contact mechanism in progenitor cell motion,^34^ paracrine mechanisms comediate MPC proliferation.^15^

The α-SMA expressions in MPCs from both patients with CAV and patients without CAV support our assumption that MPCs are able to differentiate into SMCs, which is the dominant histologic feature in CAV.^27^ Moreover, only MPCs from patients with CAV could retain, even in high passages, their α-SMA expression, clearly pointing to the contribution of MPCs to characteristic neointimal changes observed in CAV. These results are in agreement with the existing in vivo evidence that infiltrating progenitor cells are able to differentiate into SMCs^21^ and contribute to intimal hyperplasia.^35^

Several potential mechanisms could explain the higher number of MPCs in the patients with CAV than in those without CAV. One explanation might be the ongoing recruitment of MPCs to the site of intimal hyperplasia, which in turn might trigger further MPC overproduction in the bone marrow by paracrine mechanisms. A second explanation might be the inflammation that is evidently associated with CAV^36^ and triggers the production and release of cytokines and chemokines involved in MPC recruitment.^33^ A third explanation might be a compensatory reaction of bone marrow to compensate for decreased number of circulating endothelial progenitor cells in CAV.^16^ Finally, although the patient groups were matched, and although generally accepted risk factors for CAV such as acute cellular rejection were not significantly different between the patient groups, we cannot exclude the impact of such factors on MPCs in CAV. Experimental CAV models are therefore necessary for conclusive illustration of the mechanisms involved in recruitment of MPCs. These studies might then also answer the question as to how a therapeutic strategy can be developed to benefit the target MPCs in CAV treatment, particularly considering the paracrine mechanisms involved in governing MPCs.

## Figures and Tables

**Table 1:** Demographic data, clinical characteristics, and immunosuppression of study patients

Characteristics	No CAV (n = 25)	CAV (n = 25)
Age (y, mean ± SD)	65.3 ± 7	66.6 ± 8
Time from transplant (mo, mean ± SD)	111.2 ± 30.3	125.3 ± 33.2
Male sex (no.)	19 (76%)	22 (88%)
Diabetes mellitus (no.)	4 (16%)	4 (16%)
Hyperlipidemia (no.)	18 (72%)	17 (68%)
Etiology (no.)		
Ischemic cardiomyopathy	10 (40%)	10 (40%)
Dilated cardiomyopathy	15 (60%)	15 (60%)
Acute cellular rejection (no.)	2 (8%)	1 (4%)
Panel-reactive antibody level at transplant (%)	3.1	3.8
Mean ischemia time at transplant (min, mean ± SD)	175 ± 54	183 ± 51
Cytomegalovirus serostatus (no.)		
Donor positive	14 (56%)	15 (60%)
Recipient positive	12 (48%)	16 (64%)
Donor positive, recipient negative	5 (20%)	3 (12%)
Donor negative, recipient positive	3 (12%)	4 (16%)
Immunosuppression (no.)		
Cyclosporine, mycophenolate mofetil, steroids	9 (36%)	12 (48%)
Cyclosporine, everolimus, steroids	8 (32%)	7 (28%)
Tacrolimus, mycophenolate mofetil, steroids	8 (32%)	6 (24%)

Differences between groups not significant for all characteristics. *CAV,* Cardiac allograft vasculopathy.
